# BALB.NCT-*Cpox*^*nct*^ is a unique mouse model of hereditary coproporphyria

**DOI:** 10.1016/j.ymgmr.2023.100964

**Published:** 2023-03-16

**Authors:** Xiaojing Kang, Shin Shimada, Hiroki Miyahara, Keiichi Higuchi, Masayuki Mori

**Affiliations:** a Department of Aging Biology, Shinshu University Graduate School of Medicine, Science and Technology, 3-1-1 Asahi, Matsumoto 390-8621, Japan; b Division of Animal Research, Research Center for Supports to Advanced Science, Shinshu University, 3-1-1 Asahi, Matsumoto 390-8621, Japan; c Department of NeuroHealth Innovation, Institute for Biomedical Sciences, Interdisciplinary Cluster for Cutting Edge Research, Shinshu University, 3-1-1 Asahi, Matsumoto 390-8621, Japan; d Community Health Care Research Center, Nagano University of Health and Medicine, 11-1 Kawanakajimamachiimaihara, Nagano 381-2227, Japan

**Keywords:** Coproporphyria, Coproporphyrinogen oxidase, Mouse, Mutant, Pathology

## Abstract

In humans, mutations in the coproporphyrinogen oxidase (*CPOX*) gene can result in hereditary coproporphyria (HCP), characterized by high levels of coproporphyrin excretion in the urine and feces, as well as acute neurovisceral and chronic cutaneous manifestations. Appropriate animal models for comprehending the precise pathogenesis mechanism of HCP have not been reported that show similarities in terms of gene mutation, reduced CPOX activity, excess coproporphyrin accumulation, and clinical symptoms. As previously discovered, the BALB.NCT-*Cpox*^*nct*^ mouse carries a hypomorphic mutation in the *Cpox* gene. Due to the mutation, BALB.NCT-*Cpox*^*nct*^ had a drastic increase in coproporphyrin in the blood and liver persistently from a young age. In this study, we found that BALB.NCT-*Cpox*^*nct*^ mice manifested HCP symptoms. Similar to HCP patients, BALB.NCT-*Cpox*^*nct*^ excreted an excessive amount of coproporphyrin and porphyrin precursors in the urine and displayed neuromuscular symptoms, such as a lack of grip strength and impaired motor coordination. Male BALB.NCT-*Cpox*^*nct*^ had nonalcoholic steatohepatitis (NASH)-like liver pathology and sclerodermatous skin pathology. A portion of male mice had liver tumors as well, whereas female BALB.NCT-*Cpox*^*nct*^ lacked these hepatic and cutaneous pathologies. In addition, we discovered that BALB.NCT-*Cpox*^*nct*^ exhibited microcytic anemia. These results indicate that BALB.NCT-*Cpox*^*nct*^ mice serve as the suitable animal model to help gain insight into the pathogenesis and therapy of HCP.

## 1 Introduction

Coproporphyrinogen oxidase (CPOX) is an enzyme that catalyzes the conversion of coproporphyrinogen III to protoporphyrinogen IX in the heme biosynthesis pathway. In humans, mutations in the *CPOX* gene that diminish enzyme activity can be a cause of hereditary coproporphyria (HCP; OMIM #121300), a genetically dominant disease with incomplete penetrance [1]. >65 *CPOX* mutations have been identified in HCP patients [1]. The majority of the HCP patients were heterozygous for the mutant and normal *CPOX* alleles. However, the presence of a mutant gene is insufficient for disease development, and most carriers of a disease-causing *CPOX* mutation do not exhibit clinical symptoms. HCP symptoms typically manifest in the 20s or 30s, with acute attacks triggered by factors, such as fasting, alcohol, sulphonamide antibiotics, and hormones such as progesterone [2]. Increased demand for hepatic heme synthesis is thought to be the mechanism by which these factors precipitate acute attacks. Reduced CPOX activity in a *CPOX* mutation carrier causes an accumulation of coproporphyrinogen III in tissues, part of which is then autoxidized to coproporphyrin III and excreted in excess in the urine and feces. Thus, excess excretion of coproporphyrin (exceeding three times the average level) is a crucial diagnostic indicator for HCP. HCP patients exhibit clinical symptoms of both hepatic and cutaneous porphyrias. Like hepatic porphyrias, HCP manifests clinically as acute neurovisceral attacks frequently accompanied by nausea, vomiting, and constipation. The additional neurovisceral manifestations are tachycardia, hypertension, motor weakness, and seizures. Acute attacks are reversible with prompt treatment such as haem arginate infusions that reduce the overproduction of δ-aminolevulinic acid (δ-ALA) [[3], [4], [5]]. Despite this, patients experience repeated acute attacks and remissions. Also, several papers have reported HCP patients with hepatocellular carcinoma [[6], [7], [8], [9], [10]]. In the Swedish porphyria register, the fraction of HCP patients that had primary liver cancer was 1/56 [10]. Around twenty to 30% of affected patients also develop skin lesions in the sun-exposed areas of the skin. Like cutaneous porphyrias, affected individuals may experience severe pain, burning, and itching of such areas (photosensitivity). Eventually, the skin may become fragile and develop blisters with fluid. These neurovisceral and dermal findings vary significantly among patients. The precise pathogenesis mechanism of HCP is not understood.

Animal models with appropriate *Cpox* mutations are essential for comprehending the precise pathogenesis mechanism of HCP. N-ethyl-N-nitrosourea (ENU) mutagenesis has produced two strains of *Cpox* knockout mice (RBC16 and M100835) [11,12]. A nonsense nucleotide substitution produces a premature stop codon in place of tryptophan at amino acid 373 in one strain of RBC16 [11]. Homozygotes for this mutant allele are prenatally lethal due to the complete shutdown of heme biosynthesis, whereas heterozygotes were born alive. The amount of CPOX in the livers of heterozygotes was sustained at around 50% of that of wild-type mice. Intriguingly, female heterozygous RBC16 mice excreted three- to fourfold higher levels of coproporphyrin in their urine and feces than wild-type female mice. No other HCP symptoms or notable histopathology were observed in RBC16 mice of both sexes. The lack of pathology and only a moderate increase in coproporphyrin in the urine and feces of female RBC16 mice is likely because the remaining CPOX activity is sufficient to prevent coproporphyrin accumulation in the cells. Another ENU mutagenized mouse strain, M100835, has a point mutation at the acceptor site of *Cpox* intron 4 that induced an abnormal splicing event [12]. Homozygotes for the mutant allele died in utero. The HCP phenotypes of the M100835 mice have not been studied. Thus, these *Cpox* knockout mice are incompetent to be used for the exploration of the pathological mechanisms of HCP. Meanwhile, mutant *Cpox* heterozygotes exhibited microcytic anemia, caused by heme deficiency due to decreased CPOX activity [11,12].

In a previous study, we determined hereditary cataracts in the BALB.NCT-*Cpox*^*nct*^ is caused by a spontaneous missense mutation in the gene for coproporphyrinogen oxidase (*Cpox*^*nct*^ mutant allele) [13]. The mutation in BALB.NCT-*Cpox*^*nct*^ is a single amino acid substitution at the 380th position of the CPOX enzyme, from leucine to arginine (R380L). A patient with HCP heterozygous for the R391W amino acid substitution at the homologous position of the R380L substitution in BALB.NCT-*Cpox*^*nct*^ was reported [1]. The mutant CPOX-R391W was approximately 22% as active as the wild type. Intriguingly, the reduced activity of the mutant CPOX-R380L of BALB.NCT-*Cpox*^*nct*^ (∼15% of wild-type) was consistent with the human CPOX-R391W [13]. Also, concurrent with the decrease in CPOX activity, BALB.NCT-*Cpox*^*nct*^ had a drastic increase in coproporphyrin in the serum and liver, which persisted from a young age in both sexes. These results suggested that BALB.NCT-*Cpox*^*nct*^ mice exhibit clinical symptoms of HCP. In this study, this possibility was investigated by biochemical, clinical, and histopathological analysis of BALB.NCT-*Cpox*^*nct*^.

## 2 Materials and methods

### 2.1 Mice and ethical statement

Breeding pairs of inbred BALB.NCT-*Cpox*^*nct*^ congenic mice [14] that carry the mutant *Cpox*^*nct*^ gene in a homozygous state [13] were obtained from the RIKEN BRC (Tsukuba, Japan) (RBRC00422) via the National BioResource Project of MEXT/AMED (Tokyo, Japan) and subsequently bred by brother × sister mating at the Institute of Experimental Animals, Shinshu University. The BALB/c mice used as controls were purchased from Japan SLC, Inc. (Hamamatsu, Japan). These mice were maintained under clean conventional conditions at a temperature of 24 ± 2 °C under a light-controlled schedule (12-h light/dark cycle) with free access to a commercial diet (MF; Oriental Yeast Co. Ltd., Tokyo, Japan) and tap water. All experimental procedures involving mice were conducted following the Regulations for Animal Experimentation of Shinshu University and the ARRIVE guidelines. In addition, the animal protocol was reviewed by the Committee for Animal Experiments of Shinshu University and approved by the president of Shinshu University (approval number 019071).

### 2.2 Measurement of porphyrins and porphyrin precursors

Fresh urines were collected from 3-month-old male and female mice (*n* = 3) three times a day (morning, noon, and evening) for seven days and immediately frozen at −80 °C. After thawed, the urines were centrifuged at 10,000 rpm for 10 min to remove insoluble components and samples from the same individuals were collected into one tube. Blood was drawn into a syringe with 10% EDTA-2K solution as an anticoagulant from the hearts of mice under isoflurane anesthesia. Measurements of porphyrins and porphyrin precursors were outsourced to the commercial laboratory BML, Inc. (Tokyo, Japan). Uroporphyrin and coproporphyrin contents in the urine and blood and protoporphyrin contents in the blood were measured with an L-6200 HPLC system (Hitachi High-Tech, Tokyo, Japan). Porphobilinogen and δ-aminolevulinic acid (δ-ALA) in the urine was measured by a spectrophotometer (7011, Hitachi High-Tech) and a UFLC (Shimadzu, Kyoto, Japan), respectively.

### 2.3 Heart rate and blood pressure measurement

All measurements were performed during the circadian cycle's light phase. The heart rate and blood pressure of 3-month-old mice were measured using an oscillometric technique and a computerized tail-cuff system (BP-98A-L, Softron Ltd., Tokyo, Japan). Each mouse was measured three times, and the mean was used as the measured value for each mouse.

### 2.4 Forelimb grip strength test

The tail of the 3-month-old mouse that gripped a bar connected to a digital force gauge (DS2-2N, IMADA, Toyohashi, Japan) was gently pulled backward, and the maximal grip force applied until the mouse released the bar was recorded. Each mouse was measured five times, with at least one minute separating each measurement, and the average of these measurements was used as the mouse’s measured value.

### 2.5 Rotarod test

The motor coordination and balance of 3-month-old mice were analyzed using a rotarod apparatus (O'hara & Co., LTD, Tokyo, Japan). The device was programmed to accelerate the rotating speed of the rod from 4 to 40 rpm in 300 s. It was determined how long each mouse could remain on the rotating rods. Measurement was repeated three times with trials at least 15-min intertrial intervals, and the meantime was recorded as the duration observed for each mouse.

### 2.6 Histological examination

After being sacrificed by cardiac puncture under deep isoflurane anesthesia, the liver and skin from the dorsal neck, midback, and buttocks were removed from mice, formalin-fixed, paraffin-embedded, and sliced to a thickness of 4 μm for histological analysis. For the Masson-Goldner staining of liver sections, the Masson-Goldner staining kit (100485, Merck, Darmstadt, Germany) and Weigert's iron hematoxylin kit (115973, Merck) were used. After hematoxylin-eosin (HE) staining and Masson-Goldner staining, sections were viewed using an optical microscope. For transmission electron microscopy (TEM), 1–2 mm pieces of the liver were prepared, prefixed in 2.5% glutaraldehyde, and postfixed in 1% osmium tetroxide, followed by embedding in epoxy resin. The specimens were examined using a JEM-1400 electron microscope (high contrast).

### 2.7 Fluorescent immunohistochemistry

For the detection of Mallory bodies, paraffin-embedded liver sections were blocked in 5% BSA at room temperature, incubated with anti-cytokeratin 8 rabbit monoclonal antibody (1:250, ab53280, Abcam, Cambridge, UK) and anti-p62/SQSTM1 guinea pig polyclonal antibody (1:250, GP62-C, PROGEN, Heidelberg, Germany) overnight at 4 °C, and then reacted with goat anti-rabbit IgG H&L (Alexa Fluor 488) antibody (1:1000, ab150077, Abcam) and goat anti-guinea pig IgG H&L (Alexa Fluor 594) antibody (1:1000, ab150188, Abcam) for 1 h at room temperature. Cell nuclei were stained with DAPI (Invitrogen/Thermo Fischer Scientific, Waltham, MA, USA) for 10 min. Anti-Ki-67 rat monoclonal antibody (SolA15) (1:100, 14-5698-82, Invitrogen) and donkey anti-rat IgG (H + L) antibody (Alexa Fluor 488) (1:200, A-21208, Invitrogen) were employed for Ki-67 detection. Fluorescent microscopy (Zeiss Axio Observer Z1, Oberkochen, Germany) was used to obtain images.

### 2.8 Immunoblot analysis

Liver samples were homogenized in ice-cold RIPA buffer (Santa Cruz Biotechnology, Dallas, TX, USA). The homogenates were centrifuged for 15 min at 4 °C and 13,000 rpm, and the pellets were resuspended in RIPA buffer. The protein concentration was determined using a BCA Protein Assay Kit (Thermo Fisher Scientific). An aliquot equivalent to 8–10 μg protein was separated by SDS-PAGE using Tris-Tricine-SDS buffer and 8% polyacrylamide gels. The separated proteins were then transferred to a polyvinylidene difluoride membrane (Immobilon, 0.2 μm pore, (Merck Millipore, Burlington, MA, USA)) and incubated overnight at 4 °C with anti-lamin A/C (E-1) mouse monoclonal antibody (1:500, sc-376248, Santa Cruz Biotechnology) or anti-cytokeratin 8 rabbit monoclonal antibody (1:7500, ab53280, Abcam). The target proteins were detected by enhanced chemiluminescence after the membranes were then incubated with horseradish peroxidase-conjugated anti-rabbit IgG (1:4000, 7074S, Cell Signaling Technology, Danvers, MA, USA) or anti-mouse IgG (1:4000, 7076S, Cell Signaling Technology) for 1 h at room temperature.

### 2.9 Hematological and blood biochemistry tests

Under isoflurane anesthesia, blood samples were drawn from the hearts of mice into a syringe with or without 10% EDTA-2K solution as an anticoagulant. The hematological parameters were determined with an automated cell counter (XT-2000iV, Sysmex Co., Ltd., Kobe, Japan). Plasma or serum was obtained after 30 min of centrifugation at 3,000 rpm. Using the Mouse Ferritin ELISA Kit (ab157713; Abcam) and a microplate reader (SpectraMax M5), plasma ferritin levels were determined. Serum aspartate aminotransferase, alanine aminotransferase, and total bilirubin levels were determined using L-type Wako AST・J2, L-type Wako ALT・J2 (FUJIFILM Wako Pure Chemical Corporation, Osaka, Japan), and Nascauto VL T-BIL (Alfresa Pharma, Ohsaka, Japan), respectively and an auto-analyzer (Model 7180, Hitachi, Tokyo, Japan).

### 2.10 Statistical analysis

Results are expressed as mean ± SD. Two-tailed Student's *t*-test was employed for statistical analysis. *P*-value < 0.05 was considered statistically significant.

## 3 Results

### 3.1 BALB.NCT-*Cpox*^*nct*^ mice excrete excessive coproporphyrin in the urine

Blood coproporphyrin contents of male and female BALB.NCT-*Cpox*^*nct*^ were >68- and 18-fold higher, respectively than that of sex-matched BALB/c (Table 1).

**Table 1:** Comparison of porphyrins and porphyrin precursors contents in the blood and urine of 3-month-old BALB/c and BALB.NCT-*Cpox*^*nct*^ mice.

Specimen	Porphyrin and porphyrin precursor	Male		Female	
		BALB/c	BALB.NCT-*Cpox*^*nct*^	BALB/c	BALB.NCT-*Cpox*^*nct*^
Blood	Uroporphyrin (μg/dL RBC)	< 1	< 1	< 1	< 1
	Coproporphyrin (μg/dL RBC)	< 1	68.0 ± 30.5***	< 1	18.3 ± 1.2***^†^
	Protoporhyrin (μg/dL RBC)	115.7 ± 11.4	186.3 ± 51.6	131.3 ± 0.6	181.3 ± 9.3***
Urine	δ-Aminolevulinic acid (δ-ALA; mg/L)	2.0 ± 0.2	76.2 ± 10.8***	< 0.5	9.2 ± 0.5***^††^
	Porphobilinogen (mg/L)	4.4 ± 0.1	391.3 ± 61.1***	4.3 ± 0.2	30.9 ± 4.8***^††^
	Uroporphyrin (μg/g creatinine)	67.3 ± 3.5	5,595.7 ± 1,802.1**	25.0 ± 5.6	820.3 ± 27.5***^†^
	Coproporphyrin (μg/g creatinine)	196.7 ± 12.9	75,491.7 ± 1,425.3***	313.0 ± 20.2	68,409.0 ± 1,595.6***^†^

All data are expressed as mean ± SD (*n* = 3).

**P < 0.01, and ***P < 0.001 vs. BALB/c (Student's *t*-test).

^†^*P* < 0.05, and ^††^*P* < 0.01 vs. male BALB.NCT-*Cpox*^*nct*^ (Student's *t*-test).

**Fig. 1:**
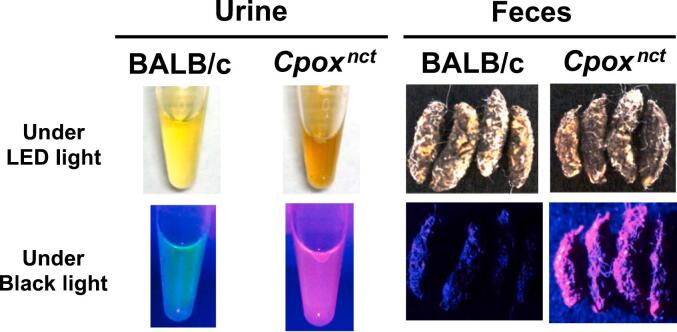
Images of the urine and feces of mice under LED light and black light.

The blood coproporphyrin contents were significantly higher in males than in females. Female but not male BALB.NCT-*Cpox*^*nct*^ had significantly higher blood protoporphyrin contents than sex-matched BALB/c. Blood uroporphyrin contents were <1 μg/dL RBC in both BALB.NCT-*Cpox*^*nct*^ and BALB/c of both sexes. Urinary coproporphyrin contents of male and female BALB.NCT-*Cpox*^*nct*^ were 385- and 219-fold higher, respectively than that of sex-matched BALB/c (Table 1). Also, urinary δ-ALA, porphobilinogen, and uroporphyrin contents were significantly elevated in BALB.NCT-*Cpox*^*nct*^ compared to BALB/c in both sexes. Urinary contents of these porphyrins and porphyrin precursors of female BALB.NCT-*Cpox*^*nct*^ were significantly lower than those of males. The color of BALB.NCT-*Cpox*^*nct*^ urine appeared reddish (Fig. 1). When irradiated with a black light (peak wavelength of 375 nm), the urine and feces of BALB.NCT-*Cpox*^*nct*^ emitted a red fluorescence suggestive of excessive porphyrins in the feces as well.

### 3.2 BALB.NCT-*Cpox*^*nct*^ mice manifest neuromuscular symptoms

The heart rate, blood pressure, grip strength, and rotarod performance of 3-month-old mice are summarized in Table 2.

**Table 2:** Heart rate, blood pressure, grip strength, and rotarod performance of BALB.NCT-*Cpox*^*nct*^ mice.

	Male		Female	
Trait	BALB/c	BALB.NCT-*Cpox*^*nct*^	BALB/c	BALB.NCT-*Cpox*^*nct*^
Heart rate (bpm)	648 ± 14	628 ± 35	579 ± 30	562 ± 21
Blood pressure (mmHg)				
Systolic	102.4 ± 5.6	108.0 ± 7.2	99.4 ± 4.3	105.8 ± 3.0*
Diastolic	71.4 ± 6.1	67.2 ± 5.0	71.1 ± 5.2	71.1 ± 3.7
Grip strength (cN)	119.2 ± 6.2	66.0 ± 5.9***	90.9 ± 5.8	57.1 ± 11.6***
Rotarod performance (*sec*)	105.0 ± 22.1	73.5 ± 9.5**	104.3 ± 17.5	73.0 ± 4.8**

All data are expressed as mean ± SD (*n* = 6).

*P < 0.05, **P < 0.01, and ***P < 0.001 vs. BALB/c (Student's *t*-test).

The heart rate of BALB.NCT-*Cpox*^*nct*^ was like that of BALB/c of the same age and sex. Female BALB.NCT-*Cpox*^*nct*^ had significantly higher systolic blood pressure than female BALB/c (+6.4 mmHg; *P* < 0.05). In contrast, male BALB.NCT-*Cpox*^*nct*^ had a systolic blood pressure comparable to male BALB/c. The diastolic blood pressure of BALB.NCT-*Cpox*^*nct*^ was comparable to that of BALB/c of the same age and sex. Forelimb grip strength of female and male BALB.NCT-*Cpox*^*nct*^ was 63% (*P* < 0.001) and 55% (*P* < 0.001) of age- and gender-matched BALB/c, respectively. Rotarod performance of BALB.NCT-*Cpox*^*nct*^ was significantly lower than that of age- and gender-matched BALB/c, indicating that BALB.NCT-*Cpox*^*nct*^ had impaired motor coordination.

### 3.3 Male BALB.NCT-*Cpox*^*nct*^ mice exhibit NASH-like pathology in the liver

**Fig. 2:**
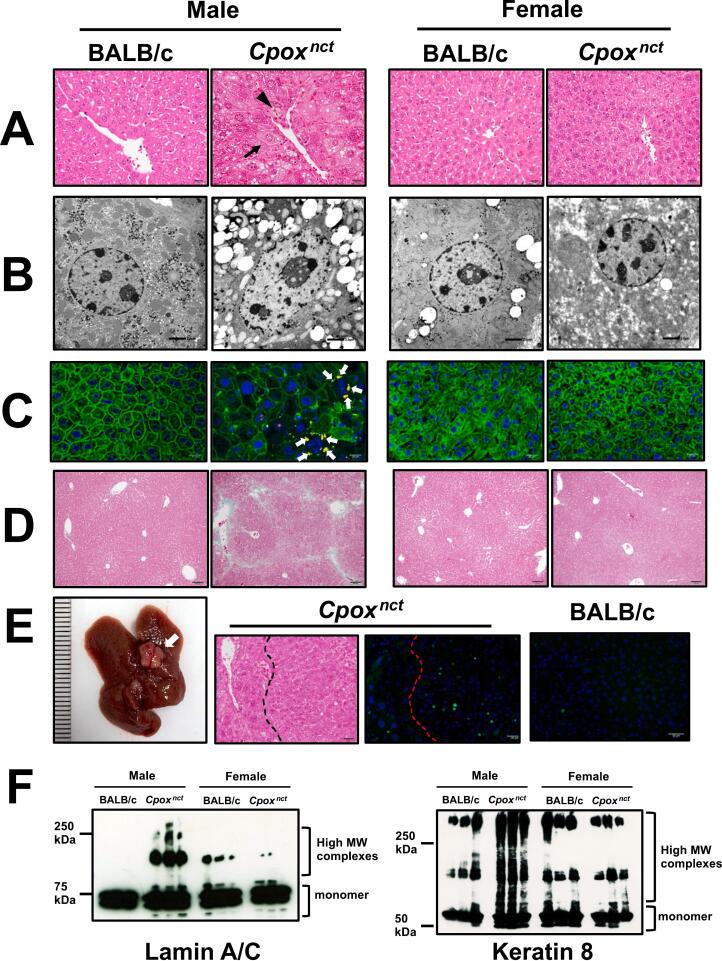
Male BALB.NCT-*Cpox*^*nct*^ mice exhibit NASH-like pathology in the liver. **A:** Light microscopic images of the HE-stained liver of 3-month-old mice. Nuclear pseudoinclusion (arrow) and pigment-filled Kupffer cell aggregates (arrowhead) are indicated. Magnification is 400×. Scale bars: 20 μm. **B:** TEM images of hepatocyte nucleus of 3-month-old mice. Magnification is 2000×. Scale bars: 2 μm. **C:** Double immunofluorescence staining of the liver of 15-month-old mice for keratin 8 (green) and p62 (red). The cell nuclei are stained blue with DAPI. Mallory-Denk bodies (overlapping yellow fluorescence) are indicated by white arrows. Magnification is 400×. Scale bars: 20 μm. **D:** Light microscopic images of the Masson-stained liver of 9-month-old mice. Magnification is 100×. Scale bars: 100 μm. **E:** Gross appearance of the liver of a 9-month-old male BALB.NCT-*Cpox*^*nct*^ mouse (**left panel**). A tumor is indicated by an arrow. HE-stained section of the tumor (right area of the broken line) and neighboring region (**middle panel**). Immunofluorescence staining of the tumor section of 9-month-old male mice for Ki-67 (green) (**right two panels**). The cell nuclei are stained blue with DAPI. Magnification is 200×. Scale bar: 50 μm. **F:** Immunoblot images of lamin A/C and keratin 8 in the liver of 3-month-old mice.

Under a light microscope, male BALB.NCT-*Cpox*^*nct*^ liver exhibited severe histopathologic changes, but not female ones. In the pericentral and midlobular regions of 3-month-old males, hematoxylin-eosin (HE) staining revealed fatty changes, hepatocyte hypertrophy, and karyomegaly (Fig. 2A). These conditions expanded gradually with age to the whole lobules of liver (Supplementary Fig. S1). Also observed were nuclear pseudoinclusions (invaginations of cytoplasm into the nucleus) in hepatocytes. Transmission electron microscopy demonstrated the expansion and deformation of the hepatocyte nucleus (Fig. 2B). Massive pigment-filled Kupffer cell aggregates were also observed in male BALB.NCT-*Cpox*^*nct*^. This transformation was most noticeable in the pericentral regions (Fig. 2A).

Mallory–Denk bodies (MDBs), a kind of hepatocellular inclusions, play an important role as histological and potential progression markers in several liver diseases, including alcoholic and nonalcoholic steatohepatitis, hepatocellular carcinoma, chronic cholestasis [15,16]. We employed a sensitive double immunoflurescence method with anti-keratin 8 and anti-p62 antibodies to detect MDBs [17] and revealed the presence of MDBs in the liver of male BALB.NCT-*Cpox*^*nct*^ mice. MDBs were only observed sporadically in the liver of 3–9-month-old BALB.NCT-*Cpox*^*nct*^ but were abundant in 12–18-month-old ones (Fig. 2C and Supplementary Fig. S2). Masson-Goldner staining of the liver revealed perivenular fibrosis in male BALB.NCT-*Cpox*^*nct*^ (Fig. 2D). The perivenular fibrosis appeared at three months of age and progressed with age, with the formation of fibrosis bridging beginning at nine months of age (Supplementary Fig. S3). These histopathological changes were not observed in 1-month-old mice but were observed in all males examined older than three months (Table 3). Additionally, one in nine males aged nine months and four of nine males aged 12–18 months had liver tumors (Fig. 2E). In tumor sections, numerous cells were positive for the proliferation marker Ki-67. These histopathological alterations resembled those observed in NASH. Immunoblot analysis revealed aggregates and polymerized forms of intermediate filament proteins lamin A/C and keratin 8, in the liver of male BALB.NCT-*Cpox*^*nct*^ (Fig. 2F). Consistent with the histopathological and biochemical findings, serum alanine aminotransferase (ALT) and aspartate aminotransferase (AST) levels as indicators of liver injury were significantly elevated in male BALB.NCT-*Cpox*^*nct*^ compared to male BALB/c after three, and six months of age, respectively, whereas those in female BALB.NCT-*Cpox*^*nct*^ mice at ages 3 and 9 months were comparable to those of age- and sex-matched BALB/c mice (Table 3). The total serum bilirubin level of BALB.NCT-*Cpox*^*nct*^ mice fell within the normal range, indicating that they did not suffer from cholestasis.

**Table 3:** Incidence of pathological change in the liver of BALB/c-*Cpox*^*nct*^ mice.

BALB.NCT-*Cpox*^*nct*^									
	Male					Female			
Age (month)	Incidence	ALT (IU/L)	AST (IU/L)	Total bilirubin (mg/dL)		Incidence	ALT (IU/L)	AST (IU/L)	Total bilirubin (mg/dL)
1	0/9 (0%)	31.0 ± 4.4	85.3 ± 17.2	0.04 ± 0.02		0/8 (0%)	ND	ND	ND
3	12/12 (100%)	120.0 ± 38.2*	84.0 ± 5.6	0.06 ± 0.01		0/8 (0%)	28.7 ± 3.1	79.7 ± 32.5	0.03 ± 0.01
6	6/6 (100%)	519.3 ± 284.2*	249.0 ± 65.9*	0.11 ± 0.03		0/3 (0%)	ND	ND	ND
9	9/9 (100%)	344.3 ± 8.0***	166.3 ± 18.8**	0.10 ± 0.03		0/11 (0%)	34.7 ± 9.0	69.7 ± 3.2	0.04 ± 0.01
12~18	9/9 (100%)	315.7 ± 107.0**	198.7 ± 67.4*	0.07 ± 0.02		0/15 (0%)	ND	ND	ND

BALB/c									

	Male					Female			

Age (month)	Incidence	ALT (IU/L)	AST (IU/L)	Total bilirubin (mg/dL)		Incidence	ALT (IU/L)	AST (IU/L)	Total bilirubin (mg/dL)

1	0/3 (0%)	26.7 ± 3.1	94.3 ± 17.0	0.03 ± 0.01		0/3 (0%)	ND	ND	ND
3	0/6 (0%)	28.3 ± 1.5	69.0 ± 29.7	0.05 ± 0.01		0/5 (0%)	26.0 ± 1.7	93.0 ± 37.0	0.05 ± 0.02
6	0/4 (0%)	55.3 ± 21.6	130.0 ± 14.5	0.11 ± 0.03		0/3 (0%)	ND	ND	ND
9	0/3 (0%)	27.3 ± 2.3	70.0 ± 20.5	0.06 ± 0.01		0/3 (0%)	25.3 ± 2.1	104.7 ± 43.7	0.07 ± 0.01
12~18	0/4 (0%)	25.7 ± 2.5	53.7 ± 7.8	0.08 ± 0.02		0/9 (0%)	ND	ND	ND

ALT, AST, and total bilirubin data are expressed as mean ± SD (*n* = 3).

*P < 0.05, **P < 0.01, and ***P < 0.001 vs. BALB/c (Student's *t*-test).

### 3.4 Male BALB.NCT-*Cpox*^*nct*^ mice exhibit sclerodermatous skin pathology

**Fig. 3:**
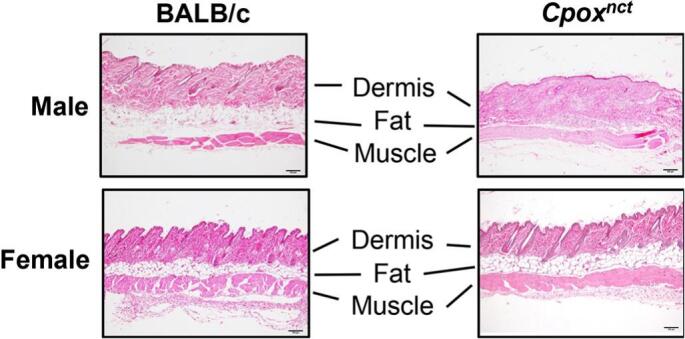
Male BALB.NCT-*Cpox*^*nct*^ mice exhibit sclerodermatous skin pathology. Light microscopic images of the HE-stained skin of 3-month-old mice. Magnification is 100×. Scale bars: 100 μm.

The subcutaneous fatty tissue of the HE-stained male BALB.NCT-*Cpox*^*nct*^ skin was often discernible and extremely thin (Fig. 3). Also, slight thickening and fibrosis of the dermis were observed. Some males older than three months exhibited these sclerodermatous changes, whereas no such alterations were observed in females (Table 4). However, the extent of these changes was highly variable between individuals and regions of skin on the same individual (Supplementary Fig. S4 and Supplementary Table S1).

**Table 4:** Incidence of histopathological changes in the skin of BALB.NCT-*Cpox*^*nct*^ mice⁎.

	BALB/c		BALB.NCT-*Cpox*^*nct*^	
Age (month)	Male	Female	Male	Female
1	0/3 (0%)	0/3 (0%)	0/9 (0%)	0/8 (0%)
3	0/6 (0%)	0/5 (0%)	4/12 (33%)	0/8 (0%)
6	0/4 (0%)	0/3 (0%)	2/6 (33%)	0/3 (0%)
9	0/3 (0%)	0/3 (0%)	7/9 (78%)	0/11 (0%)
12–18	0/4 (0%)	0/9 (0%)	6/9 (67%)	0/15 (0%)

⁎ Mice were judged to have a lesion if at least one of the three skin specimens exhibited the sclerodermatous pathology (Supplementary Table S1).

### 3.5 BALB.NCT-*Cpox*^*nct*^ mice exhibit microcytic anemia

The data in Table 5 pertain to the hematology of the peripheral blood of 3-month-old mice. BALB.NCT-*Cpox*^*nct*^ had significantly lower levels of hemoglobin (HGB), hematocrit (HCT), mean corpuscular volume (MCV), and mean corpuscular hemoglobin (MCH) versus BALB/c matched for age and gender. In addition, the coefficient of variation for RBC distribution width (RDW-CV) was significantly greater in BALB.NCT-*Cpox*^*nct*^ compared to BALB/c. The plasma ferritin levels of male BALB.NCT-*Cpox*^*nct*^ were significantly higher compared to male BALB/c, whereas no significant strain differences were observed for the female ferritin levels. These results indicated that BALB.NCT-*Cpox*^*nct*^ had microcytic anemia.

**Table 5:** Comparison of hematological parameters and plasma ferritin levels between 3-month-old BALB/c and BALB.NCT-*Cpox*^*nct*^ mice.

Parameters	Male		Female	
	BALB/c	BALB.NCT-*Cpox*^*nct*^	BALB/c	BALB.NCT-*Cpox*^*nct*^
WBC (x 10^9^/L)	2.49 ± 0.52	2.78 ± 0.60	2.51 ± 1.10	2.73 ± 1.19
RBC (x 10^12^/L)	9.67 ± 0.55	9.68 ± 0.28	9.41 ± 0.42	9.64 ± 0.62
HGB (g/dL)	15.3 ± 1.0	12.4 ± 0.9***	15.5 ± 0.9	13.6 ± 0.8**
HCT (%)	43.8 ± 1.6	35.3 ± 2.9***	42.7 ± 2.1	39.1 ± 1.3**
MCV (fL)	45.0 ± 0.9	38.5 ± 0.8***	44.5 ± 0.6	38.9 ± 0.5***
MCH (pg)	15.6 ± 0.2	13.6 ± 0.2***	15.8 ± 0.4	14.1 ± 0.2***
MCHC (g/dL)	34.8 ± 1.1	35.3 ± 1.1	35.5 ± 1.3	36.3 ± 0.6
RDW-CV (%)	23.3 ± 1.5	27.9 ± 0.8***	22.5 ± 0.9	28.7 ± 1.6***
PLT (x 10^4^/uL)	122 ± 9	131 ± 24	115 ± 6	118 ± 7
Plasma ferritin (ng/ml)	292 ± 17	439 ± 69***	361 ± 90	290 ± 42

All data are expressed as mean ± SD (n = 6).

**P < 0.01, and ***P < 0.001 vs. BALB/c (Student's *t*-test).

WBC, white blood cell; RBC, red blood cell; HGB, hemoglobin; HCT, hematocrit; MCV, mean corpuscular volume; MCH, mean corpuscular hemoglobin; MCHC, mean corpuscular hemoglobin concentration; RDW-CV, red blood cell distribution width - coefficient of variation; PLT, platelet.

## 4 Discussion

In this study, we demonstrated that BALB.NCT-*Cpox*^*nct*^ mice carrying a hypomorphic mutation in the *Cpox* gene presented HCP symptoms. Most notably, the symptoms included NASH-like liver pathology and sclerodermatous skin pathology. Curiously, however, these symptoms were observed only in males. Overall, the BALB.NCT-*Cpox*^*nct*^ showed both similarities and differences with HCP patients (Table 6). Additionally, we discovered that BALB.NCT-*Cpox*^*nct*^ had microcytic anemia.

**Table 6:** Comparison of features of HCP patients and BALB.NCT-*Cpox*^*nc*t^ mice.

Feature	HCP patients	BALB.NCT-*Cpox*^*nct*^ mice
CPOX activity	1–67% of the normal	∼15% of wild type
Mode of action of mutant alleles	Dominant with incomplete penetrance	Recessive
Disease onset	Acute (triggered by exogenous factors)	Chronic (spontaneous)
Excretion of excessive coproporphyrin, δ-ALA, and porphobilinogen in the urine	Yes	Yes
Tachycardia	Yes (highly variable)	No
Hypertension	Yes (highly variable)	Only in female
Muscle weakness	Yes (highly variable)	Yes
Motor weakness, impaired motor coordination	Yes (highly variable)	Yes
Hepatic pathology	Higher risk of primary cancer	NASH-like changes and tumor formation in male
Skin pathology	Sun-exposed area may become fragile and develop fluid-filled blisters (highly variable)	Scleroedematous pathology in male
Gender bias	Attacks are more common in women than in men	Hepatic and skin pathologies occur only in male
Microcytic anemia	Not reported	Yes
Cataracts	Not reported	Yes

To the best of our knowledge, BALB.NCT-*Cpox*^*nct*^ is the only model mouse that spontaneously manifests severe coproporphyria phenotypes. BALB.NCT-*Cpox*^*nct*^ mice exhibit the phenotypes supposedly because CPOX activity in the mice is sufficiently low, and a high coproporphyrin level persists systemically from a young age [13]. Presumably, the remaining CPOX activity in BALB.NCT-*Cpox*^*nct*^ mice (around 15% of wild-type) [13] would be sufficient to prevent embryonic death and permit postnatal growth and reproduction. However, even under normal physiological conditions, this CPOX activity would be insufficient for the conversion of coproporphyrinogen III to protoporphyrinogen IX, resulting in the systemic accumulation of coproporphyrinogen. An HCP patient due to a homozygous arginine-to-tryptophan substitution (R231W) mutation of CPOX was reported [18,19]. The onset of HCP symptoms in this patient was unusually early in childhood. She exhibited hypertrichosis and skin pigmentation affecting the face and dorsum of both hands when she was four years old. At 10 years, she was admitted to the hospital for persistent vomiting, abdominal pain, and constipation, and diagnosed with HCP. The lymphocyte CPOX activity of the patient was only 2% of normal. The patient's urinary levels of δ-ALA, porphobilinogen, and coproporphyrin were 19, 36, and 151 times higher than the normal mean or upper normal limits [18,20]. Thus, although the position of amino acid substitution in CPOX is different, the pathophysiological condition of BALB.NCT-*Cpox*^*nct*^ mice appears comparable to that of this HCP patient.

The most notable finding in this study was that male BALB.NCT-*Cpox*^*nct*^ presented NASH-like pathology and tumor formation in the liver. As far as we know, there have been no reports of NASH-like changes in HCP patients. HCP is clinically similar to other acute hepatic porphyrias: intermittent acute porphyria, variegate porphyria, and δ-aminolevulinic acid dehydratase porphyria, which are caused by mutations in the genes for porphobilinogen deaminase, protoporphyrinogen oxidase, and δ-aminolevulinic acid dehydratase, respectively. The risk of primary liver cancer in patients with acute hepatic porphyrias has been confirmed (incidence = 1.5–35%) [[6], [7], [8], [9], [10],[21], [22], [23], [24]]. Similarly in mice, protoporphyrinogen oxidase (PPO)-inhibiting herbicides cause accumulation of protoporphyrin IX in tissues, especially the liver. Accordingly, long-term administration of these herbicides to mice induces hepatocellular adenoma and carcinomas [[25], [26], [27], [28], [29]].

With regards to the mechanisms for the development of liver lesions in male BALB.NCT-*Cpox*^*nct*^, immunoblot analysis of the liver revealed aggregates and polymerized forms of cellular proteins in males. It has been demonstrated that protein aggregations of the nuclear intermediate filament (IF) protein (lamin) and the cytoplasmic IF protein (keratin 8) are formed in protoporphyrinogen IX-mediated liver injury [30,31]. In addition, the pellet fractions of HepG2 cell-free extracts treated with coproporphyrin have the potential to mediate protein aggregation [30]. IF proteins, belonging to major cytoskeletal protein families, serve important roles in the maintenance of structural integrity and cellular homeostasis [32,33]. The nuclear lamin IFs, as the main component of the nuclear lamina, play major roles in maintaining structural integrity, gene transcription, and protein transportation [34,35]. Keratin 8 forms IFs, and not only maintains cellular structural integrity but also functions in signal transduction and cellular differentiation. It has been demonstrated that coproporphyrin binds proteins possibly through the deprotonation of the carboxylate moiety of the propionic acid side chain, and leads, in the presence of oxygen, to protein oxidation and aggregation in the liver of these porphyria model mice [9,30,31,36]. Thus, the accumulation of protein aggregates and polymers in the cells would disturb cellular morphology and function, and lead to the development of various pathological conditions in the liver of male BALB.NCT-*Cpox*^*nct*^. To clarify the role of accumulated coproporphyrin and/or porphyrin precursors in developing hepatic pathology, it is necessary to conduct additional research on BALB.NCT-*Cpox*^*nct*^.

In the skin of male BALB.NCT-*Cpox*^*nct*^, sclerodermatous pathology was also observed. According to our knowledge, scleroderma complications in HCP patients have not been reported. Some patients with porphyria cutanea tarda, caused by mutations in the uroporphyrinogen decarboxylase gene, exhibit similar photosensitivity, and fragility, leading to repeated blistering and fissuring that progresses to fibrosis and becoming sclerodermatous in the late stages [37].

Curiously, the manifestation of the NASH-like and sclerodermatous pathologies was restricted to males of BALB.NCT-*Cpox*^*nct*^. The reason for the absence of the pathologies in female BALB.NCT-*Cpox*^*nct*^ is not clear. Intriguingly, urinary levels of δ-ALA, porphobilinogen, uroporphyrin, and coproporphyrin of female BALB.NCT-*Cpox*^*nct*^ were considerably lower than those of males (Table 1). These levels might be insufficient to develop pathological changes. Further studies would be required to determine whether this is the reason for the absence of symptoms in females as well as females have lower porphyrin precursor levels despite having the identical *Cpox*^*nct*^ mutation. The elucidation of the mechanisms responsible for the suppression of hepatic and cutaneous pathologies in female BALB.NCT-*Cpox*^*nct*^ should lead to the development of new effective therapies for HCP. In contrast, both female and male BALB.NCT-*Cpox*^*nct*^ mice exhibited a decline in forelimb grip strength and deterioration of motor coordination. The pathogenesis of these neuromuscular symptoms may differ from that of liver and skin pathogenesis. Neurovisceral symptoms of hepatic porphyrias are considered to be related to the accumulation of δ-ALA [38,39]. The urine δ-ALA contents in female BALB.NCT-*Cpox*^*nct*^ was 9.2 mg/L, which was equivalent to the levels in symptomatic acute intermittent porphyria patients [40]. This may be the reason why female BALB.NCT-*Cpox*^*nct*^ also presented neuromuscular symptoms.

Both sexes of BALB.NCT-*Cpox*^*nct*^ mice spontaneously developed microcytic anemia. The magnitude of hematological parameter changes was comparable to that of heterozygous *Cpox* mutant mice [11,12]. Male but not female BALB.NCT-*Cpox*^*nct*^ had elevated peripheral blood ferritin levels. The high blood ferritin levels of male BALB.NCT-*Cpox*^*nct*^ may result from the release of ferritin from damaged hepatocytes [41].

## 5 Conclusion

BALB.NCT-*Cpox*^*nct*^ mice spontaneously exhibit hereditary coproporphyria phenotypes. BALB.NCT-*Cpox*^*nct*^ should be useful to elucidate the precise pathogenesis mechanisms of HCP. Additionally, BALB.NCT-*Cpox*^*nct*^ should help validate new HCP treatments.

## Funding

This work was supported in part by Grants-in-Aid for Scientific Research (C) from the 10.13039/501100001700Ministry of Education, Culture, Sports, Science and Technology, Japan [grant number 19K06455] to M.M, and Japan Science and Technology Agency SPRING, 10.13039/501100012314Shinshu University [grant number JPMJSP2144] to X.K.

## Author contributions

**Xiaojing Kang:** Funding acquisition, Methodology, Formal analysis, Investigation, Visualization, Writing – original draft. **Shin Shimada:**Methodology, Formal analysis, Investigation. **Hiroki Miyahara:** Project administration, Supervision, Writing – review & editing.**Keiichi Higuchi:** Project administration, Supervision, Writing – review & editing. **Masayuki Mori:** Conceptualization, Project administration, Supervision, Funding acquisition, Resources, Methodology, Formal analysis, Investigation, Validation, Visualization, Writing – review & editing.

## Declaration of Competing Interest

The authors have no conflict of interest.

## Data Availability

Data will be made available on request.

## Appendix A Supplementary data

Supplementary material Image 1
