# Supplementary material for: BALB.NCT-Cpoxnct is a unique mouse model of hereditary coproporphyria
Source: Mol Genet Metab Rep. 2023 Mar 16;35:100964. doi: 10.1016/j.ymgmr.2023.100964 (PMC10036863; doi:10.1016/j.ymgmr.2023.100964)
Supplement: Supplementary file 1 — Supplementary material [file mmc1.pdf]

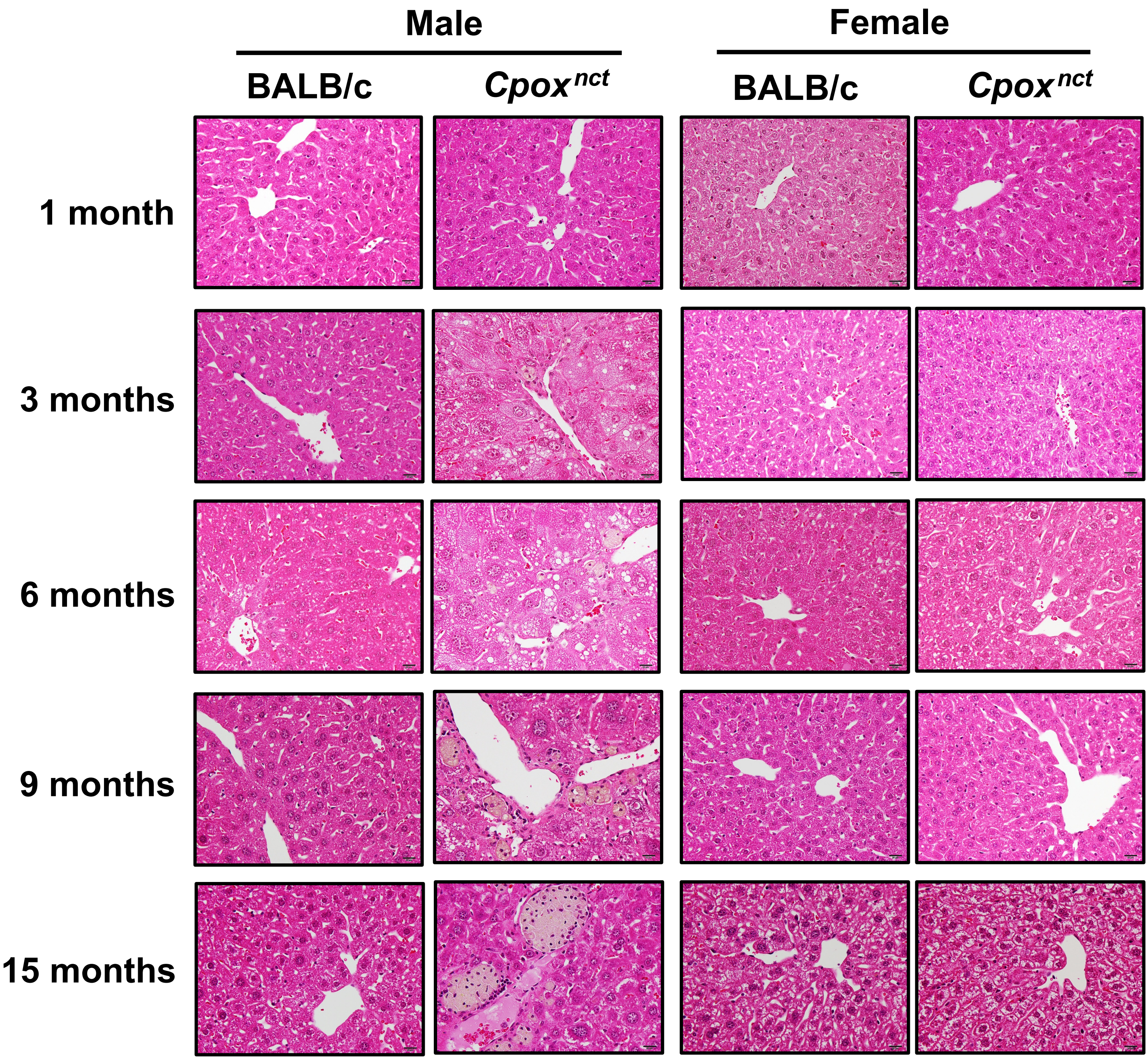

Supplementary Fig. S1. Light microscopic images of the HE-stained liver of mice of different ages. Magnification is 400×. Scale bars: 20 μm.

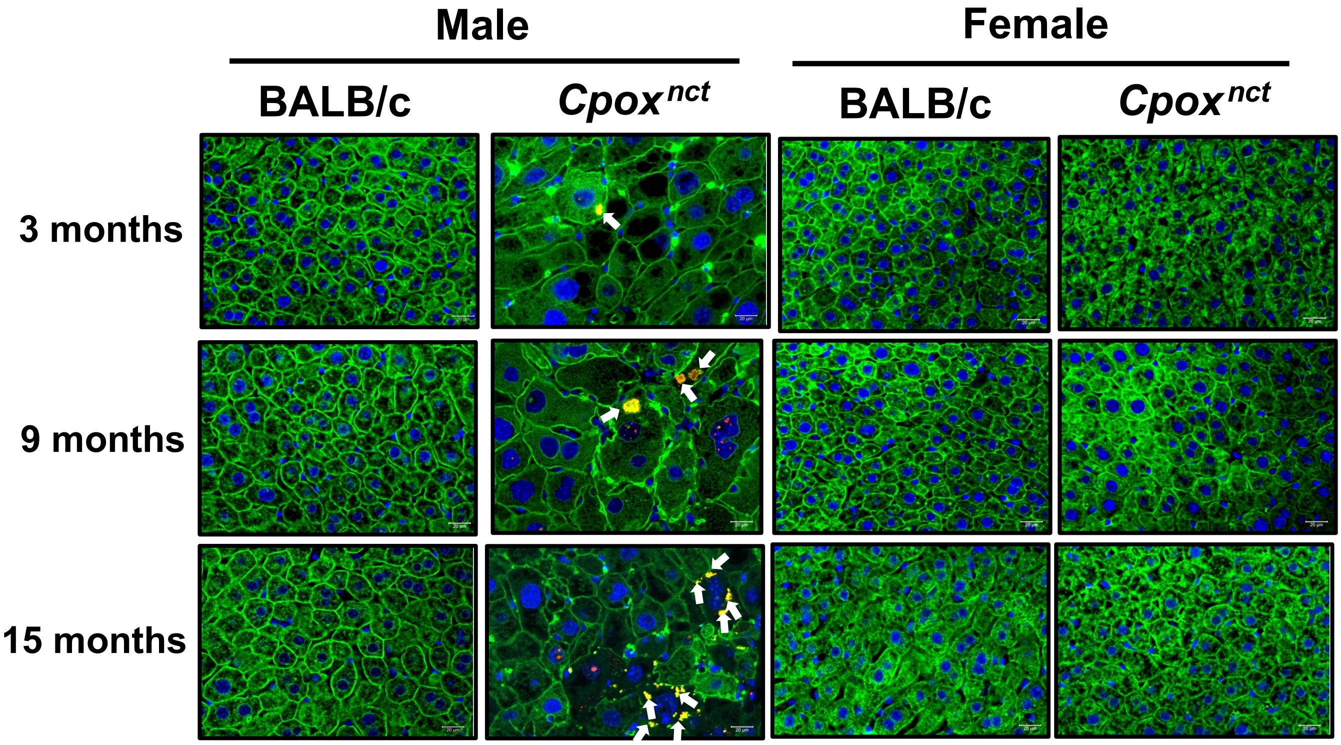

Supplementary Fig. S2. Double immunofluorescence staining of the liver of mice of different ages for keratin 8 (green) and p62 (red). The cell nuclei are stained blue with DAPI. Mallory bodies are indicated by arrows. Magnification is 400×. Scale bars: 20 μm.

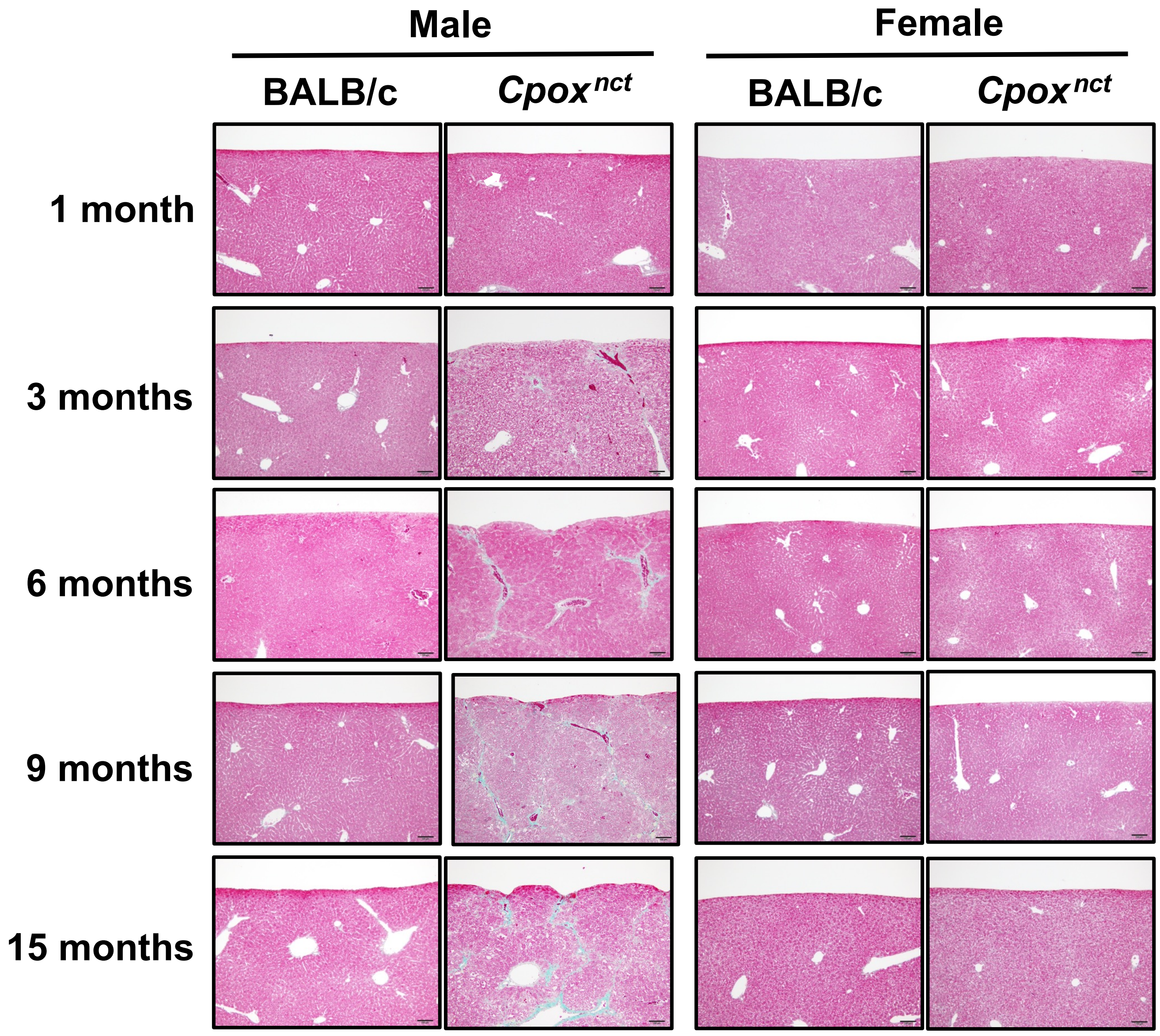

Supplementary Fig. S3. Light microscopic images of the Masson-Goldner stained liver of mice of different ages. Magnification is 100×. Scale bars: 100 µm.

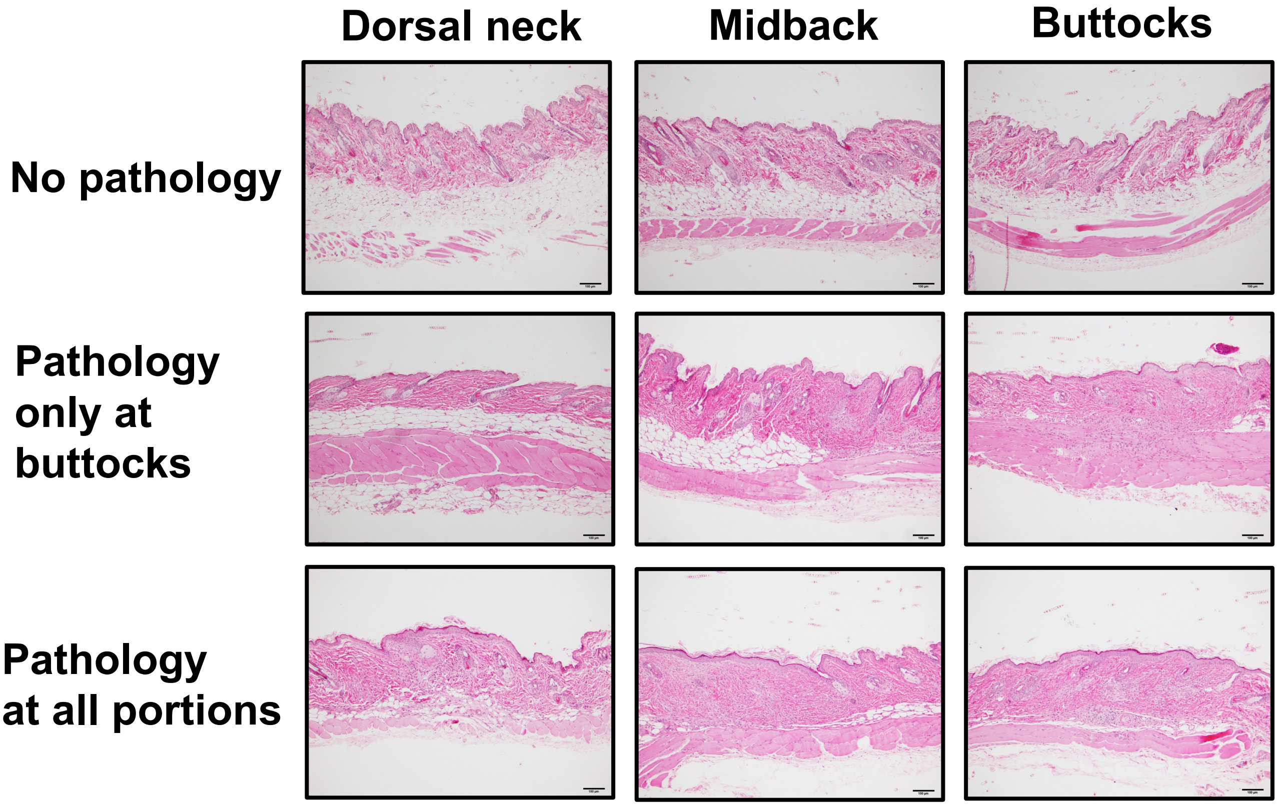

Supplementary Fig. S4. Representative images of the HE-stained skin of 3-month-old male BALB.NCT-*Cpox*^{nct} mice with different severity of sclerodermatous pathology. Magnification is 100×. Scale bars: 100 μm.

Supplementary Table S1. Presence (+) or absence (-) of sclerodermatous pathology in the skin of male BALB.NCT-*Cpox* ^{nct} mice of different ages.

1-month-old (*n* = 9)			3-month-old (*n* = 12)			6-month-old (*n* = 6)			9-month-old (*n* = 9)			12~18-month-old (*n* = 9)		
Dorsal neck	Midback	Buttock	Dorsal neck	Mid-back	Buttock	Dorsal neck	Midback	Buttock	Dorsal neck	Mid-back	Buttock	Dorsal neck	Midback	Buttock
-	-	-	-	+	+	-	-	-	+	+	+	-	+	+
-	-	-	-	+	+	+	+	+	-	+	+	-	+	+
-	-	-	-	-	-	-	-	-	-	-	-	-	-	-
-	-	-	-	-	-	-	-	-	-	-	+	+	+	+
-	-	-	+	+	+	-	-	+	-	-	-	-	-	+
-	-	-	-	-	-	-	-	-	-	+	+	-	-	+
-	-	-	-	-	-				+	+	+	-	-	-
-	-	-	-	-	-				-	+	-	-	+	+
-	-	-	-	-	-				-	-	+	-	-	-
			-	-	+									
			-	-	-									
			-	-	-									
